# Clinical Research of Mortality in Emergency Air Medical Transport

**DOI:** 10.1155/2014/767402

**Published:** 2014-08-04

**Authors:** Wan-Lin Chen, Hon-Ping Ma, Chih-Hsiung Wu, Hung-Yi Chiou, Yun Yen, Wen-Ta Chiu, Shin-Han Tsai

**Affiliations:** ^1^Institute of Injury Prevention and Control, Taipei Medical University, Taipei 110, Taiwan; ^2^College of Public Health and Nutrition, Taipei Medical University, Taipei 110, Taiwan; ^3^International SOS, Taipei 104, Taiwan; ^4^Department of Emergency, College of Medicine and Shuang Ho Hospital, Taipei Medical University, Taipei 235, Taiwan; ^5^Department of Surgery, College of Medicine and Shuang Ho Hospital, Taipei Medical University, Taipei 235, Taiwan; ^6^Taipei Medical University, Taipei 110, Taiwan; ^7^Ministry of Health and Welfare, Taipei 115, Taiwan

## Abstract

*Introduction.* EAMT in Taiwan has experienced increasing demand in the past few years. The objective is to analyze the trend of EAMT in the past six years and mortality rate within three days of patients undergoing interfacility transport in Taiwan. * Material and Method.* We conducted a retrospective review of patients who were airlifted from remote islands to main island between 2006 and 2011. Main outcome measures are EAMT number (EAMT-*N*), EAMT per thousand population (EAMT frequency, EAMT-*F*), number of mortality (Mor-*N*), and mortality rate within three days after EAMT (Mor-*R*). * Results and Discussion.* Overall mortality rate is 7.54% in 1684 airlifted patients. Acute myocardial infarction (AMI, 26.3%) and traumatic brain injury (TBI, 25.8%) comprise the majority in diagnosis (52.1%). However, Mor-*R* in these two categories is significantly low in AMI (3.5%) and TBI (5.1%). * Conclusion.* The present study demonstrates that physician density is not related to EAMT-*N* but to physician number. As general population ages (10%), the average age of patient who underwent EAMT doubled (21%). This study also leaves room for discussion regarding futile medical care. The results can be used as a reference for increasing utilization of EAMT in current National Health Care Scheme.

## 1. Introduction

The geographical limits between Taiwan main islands and its surrounding remote islands have pushed the growing need for emergency air medical transport (EAMT) [1]. EAMT in Taiwan has been used for decades and experienced increasing demand in the past few years. Ever since the National Aeromedical Approval Center (NAAC), formerly known as National Aeromedical Consultation Center (NACC), was established on October 1, 2002, EAMT in Taiwan had started a new page. Our previous studies have shown cost-effectiveness of incorporating video-telemedicine system in patient transport from remote islands and rural areas [1–3]. However, debates persist regarding the mortality, benefit, cost-effectiveness, and safety of helicopter transport [4, 5]. Transport of patients has to remove them from the hospital setting and may put them at risks of adverse clinical outcomes associated with transport and aviation mishaps. Appropriate criteria should be in place to determine when EAMT is warranted and most likely to benefit patients. Mortality after EAMT is one of the most important indicators to evaluate effectiveness. Furthermore, mortality within three days after EAMT can be a useful indicator to evaluate effectiveness of acute management of medical conditions and patient preliminary outcome.

In the present study, we analyzed cases that underwent EAMT between 2006 and 2011 in terms of general data (demography), diagnosis, and clinical outcome. We also compared the data between different remote islands to see if there is any association between physician number and EAMT parameters.

## 2. Method

Retrospective analysis of all interfacility air medical transport flight records from 2006 to 2011 was performed. The criteria for EAMT were summarized elsewhere [1]. All materials were collected from the databank of Taiwan NAAC. Data collection included two abstractors who received training on the use of SPSS 11.0 software. The database was reviewed by the two abstractors for validity and reliability. All data before and after patient transport including patient demography, disease classification, clinical outcome, and mortality rate were entered into SPSS for coding and statistical analyses. We defined elderly patients as those who were over 60 years old. In order to analyze the relationship between EAMT-*F* and resources, primary outcome measures of the study are EAMT-*N*, EAMT per thousand population (EAMT frequency, EAMT-*F*), mortality rate within three days per thousand population (Mor-*R*), physician number (PN), and physician per thousand population (physician density, PD). The relationships of age, disease classification, and mortality rate are secondary outcome measures. Demographic information of all major remote islands was obtained from government websites. Practicing PN in the remote islands was extracted from Department of Health and Local Health Bureau Registry. The research associate in charge of this study developed the data codebook and the abstractors were blinded to the research questions being tested. This study was approved by Institutional Review Board (IRB) of Taipei Medial University.

## 3. Results

There were a total of 2201 cases between 2006 and 2011. There were 1882 requests for interfacility EAMT and 1682 patients were approved for EAMT (approval rate: 89.5%). No patients died during air medical transport. There had been no air crash during the study period. Overall mortality rate is 7.54% (range: 6.11–10.88%, Table 1).

**Figure 1:**
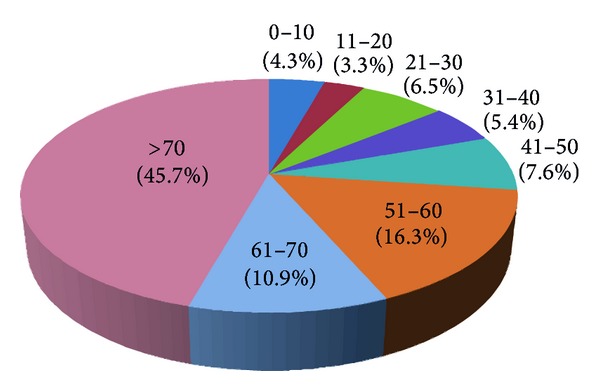
Mortality case percentage by age group.

Mean age of patients in mortality within three days group is older than that of total patient group (52.3 years versus 49.5 years; *P* = 0.06). Overall, the elderly comprised majority (56.6%) of mortality cases (Figure 1). Teenage patients only accounted for less than 4% of cases (Figure 1 and Table 1). During study period, the average age of all airlifted patients rose from 49.5 years to 59.9 years, an increase of 21%. The increased number of the elderly population among these remote islands is 10.1%. In addition, the percentage of elderly airlifted patients increased from 34.7% to 51%, a marked increase of 46.9% within the six years.

**Figure 2:**
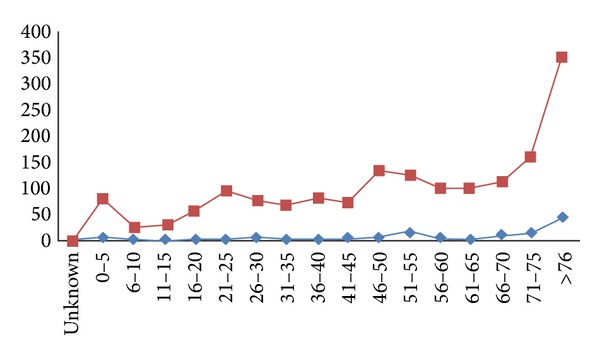
Mor-*N* further divided by age subgroup (Red: EAMT-*N*; Blue: Mor-*N*).

When we compared age distribution between all patients who underwent EAMT and all mortality cases, we noted that there is a significant association between advanced age and Mor-*N*. Mor-*N* increased remarkably in patients over 70 years old (Figure 2). The Mor-*N* of the age older than 70 years is 45.7% (Figure 1 and Table 2). The Mor-*N* within three days is almost as low in patients between 21 and 50 years old as teenage group although EMAT-*N* is more.

**Figure 3:**
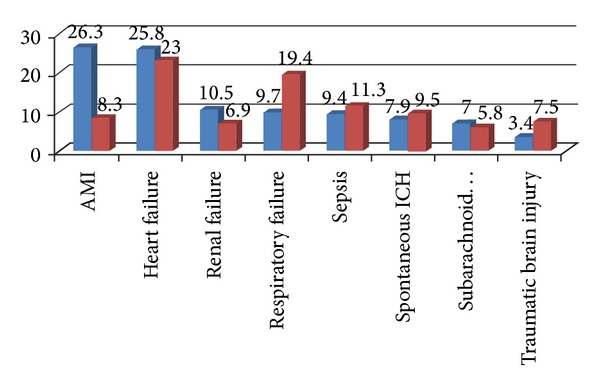
Comparison of percentage of major diagnosis in mortality cases in all (blue bar) and elderly patients (red bar).

Overall disease classification of mortality case within three days showed more medical (74.2%) than surgical conditions (25.8%). Pediatric mortality cases only accounted for less than 5% of cases. Among major diagnoses of mortality cases, acute myocardial infarction (AMI) and traumatic brain injury outnumbered other diseases (Figure 3).

**Figure 4:**
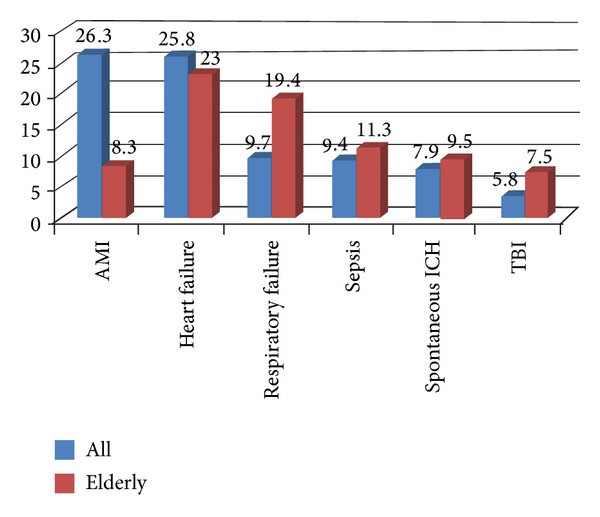
Comparison of major diagnosis in mortality cases in all (blue) and elderly patients (red).

In the elderly patient group, the percentage of major diseases differs significantly from all age group. Due to high percentage of comorbidities in the elderly group, organ failure outnumbered trauma or AMI (Figure 4).

**Figure 5:**
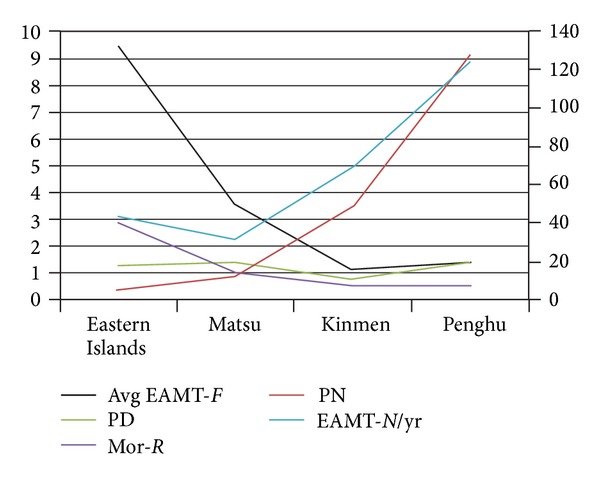
EAMT, physician number, and mortality number by region.

The discrepancies in healthcare resources between different remote islands result in some interesting findings regarding Mor-*N* and Mor-*R* that worth highlighting. Among the major remote islands, Eastern islands had both the lowest PN, and EAMT-*N* is the lowest among all remote islands, but EAMT-*F* is the highest. We found that PD has no significant difference among all remote islands. As total PN increases, average EAMT-*F* and Mor-*R* decreases. There is an association of PN and EAMT-*N* and Mor-*R* (Table 3). When total PN is approaching 50, there is a significantly lower Mor-*R* (Figure 5).

## 4. Discussion

This is the first report that there is not only no air crash in 1682 flights in six years but also no air crash in 2806 flights in ten years [1, 2]. This demonstrates effectiveness on patient safety and flight safety with NAAC mechanism. NAAC, physician-based center on a 7/24 basis, works with Central Weather Bureau, Civil Aviation Authority, and National Airborne Service Corp in a joint command center and helps to do preflight assessment with telemedicine system by physicians in NAAC and provides major contribution. Interfacility transfer is now common for a variety of critical conditions, and necessity for a higher level of care is the commonly cited justification [6]. This kind of transfer has been increasing more and more in remote islands and rural areas not only due to medical necessity but also due to personal wish, especially in National Health Care. In this study, we analyzed Mor-*N* after EAMT from 2006 to 2011. We find that the Mor-*R* is the highest and in patients aged over 70 years. The percentage between medical and surgical diseases differs significantly in the elderly (medical : surgery = 74 : 26). The ratio significantly differs from a number of reports in EAMT (medical : surgical = 61 : 39) previously.

Although the mortality rate (10.88%) was slightly higher in 2009, this higher rate was not statistically significant compared with all other years (*P* = 0.34). This is the pooled data from all regions. There had been no major change in population structure in remote islands in the study period [7]. The mortality case number in 2009 doubled compared with previous year, whereas the total case number increased only 17.3%. The reason for increased case number in 2009 is mainly respiratory complications from severe H1N1. The disproportional increase in case number of 2009 may stand for relatively high mortality rate.

Our study demonstrated that elderly comprised majority (56.6%) of mortality cases while teenage patients only accounted for the least (1.6%). This finding was similar in some studies of helicopter emergency medical services (HEMS) [8–10]. However, the present study is the first report to demonstrate that the tendency of age is a key factor of change in EAMT, not only in EAMT-*N* but also in Mor-*R*. As general population ages, the demand and need for EAMT almost doubles. Whether EAMT benefits patients in elderly group over young age group is yet to be determined [11, 12]. In Taiwan, where all population is covered by National Health Insurance, medical expense is much lower than most of developed countries. Copayment is almost always only 10% of total medical expenses. In addition, for ICU patients, copayment is waived due to the consideration of critical condition and economic ability of patient, which is equivalent to free of out-of-pocket expense. This can be the most important reason that elderly patients account for the majority of transferred and mortality cases. When comparing mortality causes between all group and elderly group, we noted that there are significant differences. In all age group, time-sensitive medical conditions, like AMI and TBI, account for the majority of EAMT-*N*. However, in the elderly group, chronic obstructive pulmonary disease (COPD) and heart failure account for the majority of EAMT-*N*. For time-sensitive diseases, timely EAMT, which provides patients with upgraded medical care, would be most beneficial. There has been accumulated evidence showing the effectiveness in these time-sensitive conditions. While acute on chronic conditions, like heart failure, the cost-effectiveness of EAMT is controversial. In National Health Insurance Scheme in Taiwan, government has to be fully responsible for EAMT, especially in remote islands. For patients who undergo EAMT and expire in Taiwan main island, the ferry back to remote islands is also subsidized by the local government with home hospice. This is another incentive that drives EAMT. The challenge is that transferring the elderly nontrauma patients may have no effect on outcomes and raise the concern of futile medical care.

Among the remote islands, Eastern Islands had the lowest PN. But when we look at PD, there was no significant difference among all remote islands. As total PN increases, EAMT-*N* and Mor-*R* decreased. There is an association of PN and EAMT-*N* and Mor-*R*. Increased PN usually increased specialists. When PN is approaching 50, the association becomes nonsignificant (Figure 4). Total PN of 50 in Kinmen is the ideal condition for both EAMT-*F* and Mor-*R*. There are two hospitals in Penghu and one in Kinmen. Lack of PN in Eastern Islands also accounted for higher EAMT-*F* and Mor-*R* than Penghu and Kinmen. In addition, average life expectancy in Eastern Islands (74.3 years) is lower than that in other remote islands (over 78.93 years, *P* = 0.07). Therefore, the present study suggests that the ideal PN plays a major role not only directly in medical care but also indirectly in public health.

In the present study, we also find that the impact of aging population on National Health Insurance is underestimated. Therefore, appropriate selection of patients by referring physicians and maintenance of good physician-patient rapport are important when the projected outcome is an issue [13, 14].

Transport of critical patients by air has become an integral part of regionalized systems of healthcare. The development of effective time-critical interventions for nontrauma patients, especially AMI and ischemic stroke, has improved the outcomes [15, 16]. In our study, AMI is one of the most common diseases transferred in our series. Given the transport distance and the need for timely intervention, transport of AMI patients would not be most beneficial. There was 46% of patients who were not quick enough to meet 90 minutes door-to-balloon time after EAMT. The level of care AMI patients need exceed the capabilities and facilities of these remote hospitals. Although the budget for purchasing the facilities and equipment of cardiac catheterization is not an issue for AMI patients, the difficulty is that the number of cases is too small to successfully recruit the specialists in local. Excessive distance and weather condition hinder transport to definitive care. For non-time-sensitive medical conditions, especially chronic diseases, Mor-*R* is high and these non-time-sensitive conditions are not good candidates for EAMT. Telemedical consultations of specialists in medical center can provide support to remote islands. Solutions to improve local healthcare capabilities include allocation of manpower, software/hardware upgrading, and incorporation of video-telemedicine system.

Limitation of this study is due to a lack of similar system as control group because no other country's healthcare system is similar to Taiwan's National Health Insurance Scheme currently although a number of countries are going to proceed with this kind of system.

## 5. Conclusion 

This is the first study to provide characteristics of patients who underwent EAMT and expired within three days after EAMT. The present study also demonstrates that the number of physicians, including the specialist deployment, is very important in reducing mortality and EAMT. There is no patient safety issue owing to NAAC gate-keeping mechanism. As general population ages, the age of patient who underwent EAMT increased and EAMT-*N* increased significantly. This study compared the different disease pattern requesting EAMT in general and elderly patients; the study also leaves room for discussion regarding futile medical care. The results can be used as a reference for increasing utilization of EAMT, improving medical quality of care, transport efficacy, adequate resource allocation, and public health.

The present study also shows the importance in the selection of patients for transport particularly when it may not bear influence on clinical outcome. This study can be used as a reference for increasing medical quality of care, transport efficacy, and adequate resource allocation, especially in national health care preparedness.

## Figures and Tables

**Table 1:** Mortality case number, total patient airlifted, and mortality rate by year.

Year	Mor-*N *	EAMT-*N *	Mor-*R* (%)	*P* value
2006	18	216	8.33	0.14
2007	18	270	6.67	0.16
2008	18	282	6.38	0.19
2009	36	331	10.88	0.07
2010	19	311	6.11	0.21
2011	18	274	6.57	0.15
Total	**127**	**1684**	**7.54**	**NA**

**Table 2:** Percentage of mortality case by age group and year.

Year	Age							
	0–10	11–20	21–30	31–40	41–50	51–60	61–70	>71
2006	0	0	0	11	11	22	16.7	39.3
2007	0	0	0	5.6	5.6	27.8	5.6	55.4
2008	0	0	11	5.6	11	11	22	39.4
2009	5.6	2.8	0	5.6	13.9	13.9	25	33.2
2010	5.3	0	5.3	0	10.5	5.3	26.3	47.3
2011	0	0	0	0	5.6	16.7	22.2	55.5
Avg	**1.8**	**0.5**	**2.7**	**4.6**	**9.6**	**16.1**	**19.6**	**45**

**Table 3:** Comparison of population, physician number, EAMT, and mortality by different remote islands.

	Eastern Islands	Matsu	Kinmen	Penghu
Avg population	4,700	9,000	60,000	90,000
Ave life expectancy	74.3	80.2	79	78.93
EAMT-*N*/yr	44	32	69	124
PN	6	13	49	128
EAMT-*F *	9.4	3.6	1.15∗	1.4∗
PD	1.3	1.4	0.8	1.4
Mor-*R *	2.8	1.1	0.6^#^	0.6^#^

^∗,#^ *P* < 0.05.
